# Controllable Edge Feature Sharpening for Dental Applications

**DOI:** 10.1155/2014/873635

**Published:** 2014-03-11

**Authors:** Ran Fan, Xiaogang Jin

**Affiliations:** State Key Lab of CAD&CG, Zhejiang University, Hangzhou 310058, China

## Abstract

This paper presents a new approach to sharpen blurred edge features in scanned tooth preparation surfaces generated by structured-light scanners. It aims to efficiently enhance the edge features so that the embedded feature lines can be easily identified in dental CAD systems, and to avoid unnatural oversharpening geometry. We first separate the feature regions using graph-cut segmentation, which does not require a user-defined threshold. Then, we filter the face normal vectors to propagate the geometry from the smooth region to the feature region. In order to control the degree of the sharpness, we propose a feature distance measure which is based on normal tensor voting. Finally, the vertex positions are updated according to the modified face normal vectors. We have applied the approach to scanned tooth preparation models. The results show that the blurred edge features are enhanced without unnatural oversharpening geometry.

## 1. Introduction

**Figure 1:**
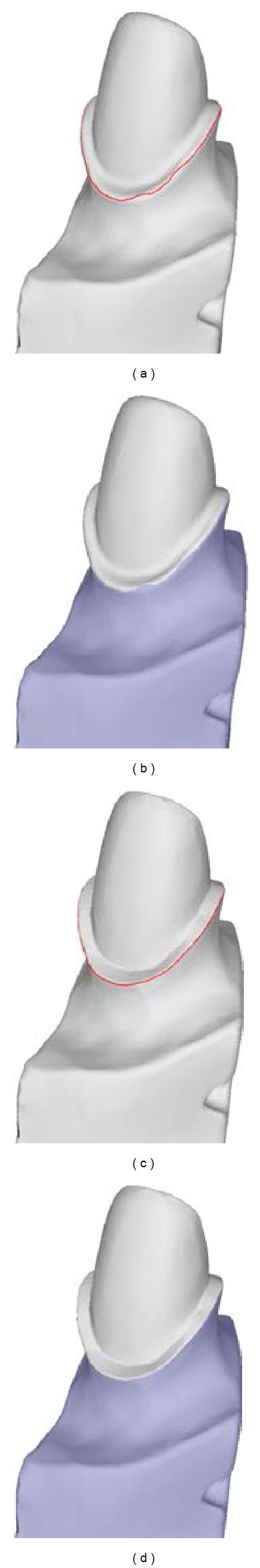
The comparison of cervical line extraction on the original tooth preparation and the sharpened model: (a) extracted cervical line on the original tooth preparation has obvious bumps due to the blurring of the edge features, (b) the segmentation along the cervical line in (a), (c) extracted cervical line on the sharpened model, and (d) the segmentation along the cervical line in (c).

Optical scanning and geometric processing are two critical techniques in dental CAD systems which are responsible for acquiring tooth shapes and designing dental appliances, respectively. Various studies have been published on building dedicated scanning systems [1, 2] and automating the procedure of generating the shapes of dental appliances [3–5]. However, there are still limitations, of which the feature blurring is a prominent one. The feature blurring problem has a significant impact on the cervical line extraction which is a necessary step in modeling various dental restorations. As shown in Figure 1(a), the original scanned tooth preparation model contains blurred feature regions, which makes the automated cervical line extraction unreliable. The problem lies in the limitations of the structured-light principle. For example, algorithms based on phase analysis [6] confine the data density according to the resolution of the projected fringes. It is difficult to be solved by improving the structured-light algorithms. As a result, geometric postprocessing is essential to further improve the quality of the scanned surfaces. As shown in Figure 1(c), by sharpening the blurred feature regions, high-quality cervical lines are obtained robustly.

Geometric filtering is a versatile tool to alter the properties of scanned surfaces represented by triangle meshes. It can make scanned surfaces more appropriate for specific visualization and shape-based product design tasks. For example, surface noise [7–10], the most common defect, can be reduced by geometric filtering, and geometric filtering-based feature enhancement can be used to exaggerate the microstructure on the artifacts surface in archeology. In order to emphasize the interesting surface attributes, a variety of filtering approaches have been developed to modify derived differential quantities instead of vertex positions. For example, Laplacian coordinate has been employed for mesh denoising and enhancing [11, 12]; curvature has been prescribed to directly control the shape of the surface in [13]. In comparison with algorithms involving second-order differential attributes, normal based filtering algorithms [14–16] are more appropriate to process anisotropic features. The reason is that the second-order differential attributes integrate characteristics in all directions so that they are not flexible to constrain anisotropic features in some directions. Although existing geometric filtering algorithms alleviate the feature blurring problem to some extent, none of them considers the degree of the sharpness. The processed edge features usually show unnatural oversharpening geometry.

In this paper, we focus on the problem of enhancing blurred edge features in a controllable manner. Specifically, the degree of the sharpness or the fillet radius is controlled to avoid oversharpening geometry. We propose a feature distance measure based on normal tensor voting to control the normal filtering process. After the filtering, the vertex positions are updated by fitting the new face normal vectors in the least square sense. In addition to geometric filtering, feature region detection is also important for solving the feature blurring problem since engineering users demand high-fidelity scanned surfaces. As a result, the featureless regions should be untouched. We consider this problem as a segmentation to avoid involving a user-defined threshold which is common in most prior researches. We adopt a graph-cut method to compute the segmentation. The main contributions of the paper contain three aspects as follows.

1. Unlike most existing mesh sharpening methods which produce oversharpening geometry benefitting high-quality visualization, the proposed mesh sharpening method, which controls the sharpness or the fillet radius of edge features, is more appropriate for designing shapes of dental appliances. The essential strategy is also applicable to process scanned models used in mechanical and arts industry.
2. We propose a feature distance measure based on normal tensor voting to control the sharpness of edge features.
3. We cast the feature region detection into a segmentation problem and solve it with a graph-cut algorithm.

The remainder of this paper is organized as follows. In Section 2, we review the most relevant previous works. Then an overview of our approach is presented in Section 3. The core algorithms of the feature region segmentation and the controllable mesh sharpening are detailed in Sections 4 and 5, respectively. After discussing the results and the applications of our approach in Section 6, we conclude the paper in Section 7.

## 2. Related Works

### 2.1. Mesh Detail Editing

Several mesh denoising algorithms adapt two-dimensional signal processing theory to filter vertex positions. Taubin [7] proposed the first low-pass filtering algorithm for mesh smoothing. Desbrun et al. [8] improved the efficiency of the filter through an implicit solver. In order to achieve feature preserving, a variety of methods employed bilateral filters [9, 10] and anisotropic diffusion [20, 21] to reduce noises in flat regions while they maintain discontinuities in high contrast regions. In contrast to directly dealing with vertex positions, several researchers [11, 14–16] found that filtering high-order differential quantities brings obvious advantages in terms of flexibility and effectiveness. Shen and Barner [14] applied the fuzzy filter on normal vectors, and Yagou et al. [16] applied the boost filter on normal vectors. Since the edge features are naturally represented as discontinuity or large variance of normal vectors, the normal vectors are appropriate for modeling sharp edge features. Su et al. [11] first filtered the Laplacian coordinates and then reconstructed vertex positions. With similar ideas, Wang et al. [12] detailed versatile effects based on filtering Laplacian coordinates. Recently, algorithms which involve explicit feature detection [22, 23] and classify vertices into feature and featureless regions have been proposed based on the idea that multiple segments with different attributes should not be blended. Different vertex groups in neighborhood structure are filtered separately.

Edge and corner features are important for CAD and sculpture models used in mechanical and arts industry. Unfortunately, the edge and corner features are commonly degenerated depending on how the models are obtained. As a result, mesh sharpening is required to reconstruct the sharp edge and corner features which do not exist in original mesh surfaces. Attene et al. [24] proposed a two-step method to repair sharp edge features for mesh surfaces extracted from volume data. Wang [17] employed an incremental filter to extend the geometry of smooth region into the feature region. Wang [25] took advantage of the bilateral filter [10] to detect and recover sharp features. Chen and Cheng [26] used a sharpness dependent filter to recover sharp structure in surface hole-filling. Chen and Cheng [26] presented a normal filtering-based algorithm to form sharp edge features. Actually, the key idea of prior algorithms is based on the assumption that the sharp features are intersections between smooth regions. Different strategies are taken to extend smooth regions to form sharp features. However, these methods inevitably produce oversharpening geometry which is undesirable for scanned mesh surfaces.

In addition to the above local methods, global optimization methods are also developed, which can take advantage of the integral property of mesh models. For example, Ji et al. [19] proposed a global optimization procedure to enhance mesh surfaces. He and Schaefer [27] proposed *L*_0_ optimization to improve the mesh quality. Although global methods provide high quality results, they require high computation time and memory footprint in general. Moreover, the local characteristics can hardly be controlled by the global methods.

### 2.2. Feature Detection

Sharp features especially edge features play an important role in structure-aware shape processing tasks. For example, in reverse engineering, mesh surfaces are separated along feature lines and fitted into surface patches. Most existing approaches focus on extracting feature lines. Rössl et al. [28] extracted feature lines using morphological operators. Yoshizawa et al. [29] detected feature lines based on the differential definition of the valleys and ridges and located the feature lines by using local surface fitting. All of the above methods are based on curvature information. In contrast, Kim et al. [30] took advantage of normal tensor voting to classify the features into different categories and grouped feature regions through *k*-means clustering in the feature space. Wang et al. [31] extended the normal tensor voting method to extract feature lines by proposing a neighbor support saliency. In this paper, feature regions are detected to reduce the amount of calculation.

## 3. Overview

**Figure 2:**
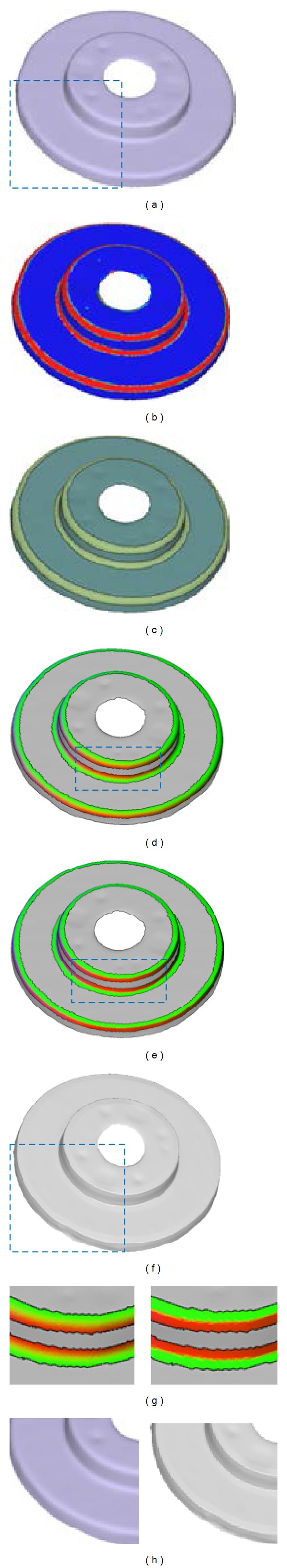
The proposed controllable mesh sharpening approach: (a) an input mechanical model in which edge features are blurred, (b) the color map of the feature distance measure, (c) feature regions which are separated via a graph-cut segmentation, (d) color map of face normal vectors before normal filtering, (e) color map of face normal vectors after normal filtering, (f) the resulting model with sharp edge features but without oversharpening geometry, (g) zoomed regions in (d) and (e), and (h) zoomed regions in (a) and (f).

The target models of our mesh sharpening algorithm are scanned surfaces produced by optical scanning systems. They commonly have a great number of triangles, which makes global approaches such as [19] unqualified. Moreover, the scanned surfaces produced by structured-light scanners can achieve accuracy of about 60 *μ*m, which makes mesh denoising unnecessary. With these considerations, the method in this paper consists of three main stages: (1) detect feature regions, (2) filter the normal vectors of triangle faces, and (3) update vertex positions according to the filtered normal vectors. Although the method in [18] has taken similar steps to sharpen mesh surfaces, the improvements of our method include two aspects: (1) we avoid user-defined thresholds through graph-cut segmentation and (2) in order to avoid oversharpening geometry, a feature distance measure quantifying the distance away from the smooth region, as illustrated in Figure 2(b), is proposed based on normal tensor analysis.

Prior feature region detection algorithms for mesh sharpening [17, 18] commonly analyze normal variance in the local neighborhood of a central face and specify a threshold to identify feature regions. This strategy does not consider the spatial coherence of the detected feature regions. In contrast, we adopt a graph-cut algorithm which involves spatial constraint as shown in Figure 2(c).

The key ideas of most effective mesh sharpening algorithms [17, 18] are similar which propagate the geometry from smooth regions to feature regions to form edge intersections. Plane fitting and skeletonisation are used in [17]; normal filtering and greedy propagation are adopted in [18]. However, these approaches inevitably produce oversharpening edge features which are unnatural for scanned surfaces. Our algorithm involves a feature distance measure to control the degree of sharpness. Figures 2(d) and 2(e) show the normal color map before and after the filtering process.  Figure 2(f) shows the final result in which the edge features are enhanced but do not suffer from oversharpening defects.

## 4. Feature Region Detection Using Graph Cuts

A given scanned surface can be represented by a triangle mesh *M*(*V*, *F*), where *V* = {*v*_*i*_ | *i* = 1,2,…, |*V*|} and *F* = {*f*_*i*_ | *i* = 1,2,…, |*F*|} are the vertices and triangle faces, respectively. Here |·| denotes the cardinality of a set. Each face *f*_*i*_ has a normal vector which is denoted by *n*_*i*_.

### 4.1. Feature Distance Metric

**Figure 3:**
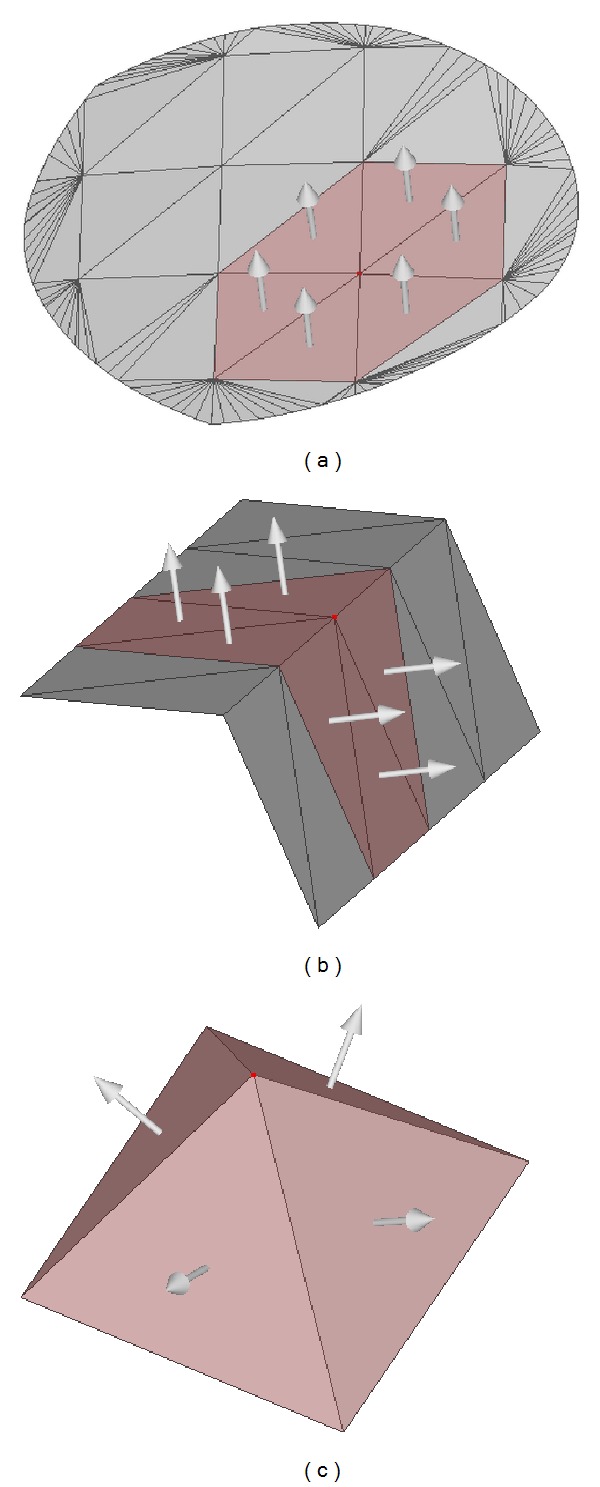
Normal tensor analysis: (a) smooth surface, *σ*_1_ = 73.7, *σ*_2_ = 0.207, and *σ*_3_ = 0.0675, (b) sharp edge feature, *σ*_1_ = 7.0, *σ*_2_ = 7.0, and *σ*_3_ = 0.0, and (c) corner feature, *σ*_1_ = 1.33, *σ*_2_ = 1.33, and *σ*_3_ = 1.33.

The normal tensor describes the local structure of a vertex of *M*. As suggested by Kim et al. [30], the normal tensor classifies local geometries into three types of features, namely, smooth surface, edge feature, and corner feature. The normal tensor at *v*_*i*_ is defined as

(1) $$\begin{array}{c} A_{v_{i}}=\underset{j\in N_{v_{i}}}{\sum }w_{j}n_{j}^{T}n_{j}, \end{array}$$

where *N*_*v*_*i*__ is the face set in the one-ring neighborhood of *v*_*i*_ and *w*_*j*_ is the weight for the covariance matrix generated by face normal vector *n*_*j*_. The difference of the definitions of normal tensor is mainly about the definition of *w*_*j*_ which is defined here as

(2) $$\begin{array}{c} w_{j}=\frac{\text{area}\left(f_{j}\right)}{\text{are}{\text{a}}_{\max}}\cdot \exp\left(-\frac{||c_{f_{j}}-v_{i}||}{\sigma /3}\right), \end{array}$$

where area(*f*_*j*_) is the area of *f*_*j*_ and area_max⁡_ is the maximum triangle area among *N*_*v*_*i*__; *c*_*f*_*j*__ is the barycenter of *f*_*j*_ and *σ* is the edge length of the bounding box including *N*_*v*_*i*__. The eigendecomposition of *A*_*v*_*i*__ uncovers the local structure of *v*_*i*_:

(3) $$\begin{array}{c} A_{v_{i}}={\left(\begin{array}{ccc} E_{1} & E_{2} & E_{3} \end{array}\right)}^{T}\left(\begin{array}{ccc} \sigma_{1} & 0 & 0\\ 0 & \sigma_{2} & 0\\ 0 & 0 & \sigma_{3} \end{array}\right)\left(\begin{array}{ccc} E_{1} & E_{2} & E_{3} \end{array}\right), \end{array}$$

where *σ*_1_ ≥ *σ*_2_ ≥ *σ*_3_ ≥ 0 are three eigenvalues of *A*_*v*_*i*__ and *E*_1_, *E*_2_, and *E*_3_ are three corresponding eigenvectors. As shown in Figure 3, the relative values of *σ*_1_, *σ*_2_, and *σ*_3_ determine the feature type in the neighborhood of *v*_*i*_.

**Figure 4:**
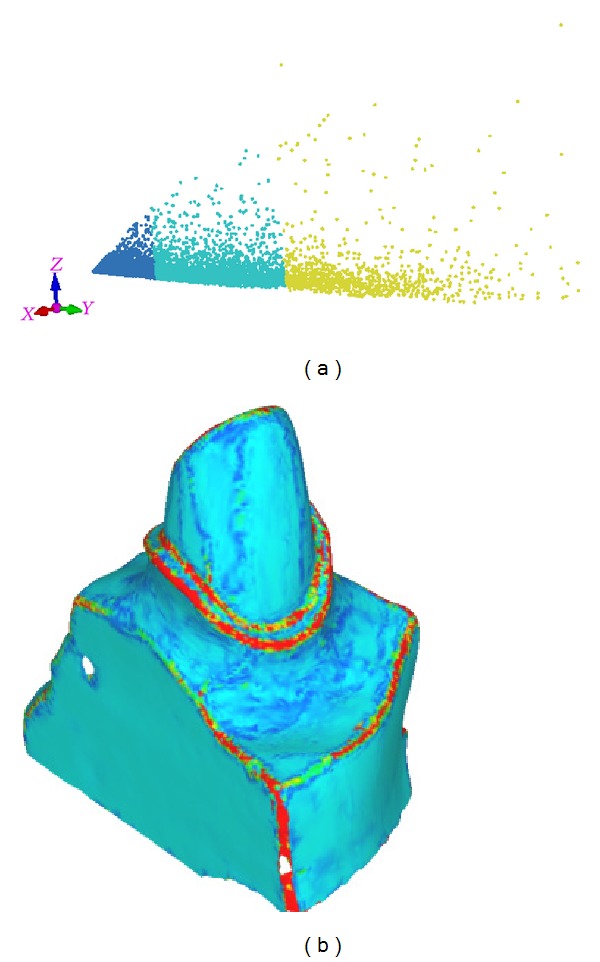
Feature distance measure: (a) *k*-means in feature space formed by eigenvectors of the normal tensors, the cluster with highest *X* component corresponds to the smooth region of *M* and (b) color map of the feature distance defined in (4).

Based on above normal tensor framework, we define a feature distance measure in feature space constructed by eigenvectors of *A*_*v*_*i*__. First, we find the feature points corresponding to smooth regions of *M* through *k*-means clustering in the feature space. As shown in Figure 4(a), after *k*-means clustering, the feature points are separated into different compact groups. The final result does not heavily depend on the parameter *k* which is chosen as 3 in this paper. The group with highest component along the largest eigenvector is denoted by the smooth set. Other feature points outside the smooth set form the feature set. In order to quantify how feature points are far away from the smooth set, the feature distance measure is defined as the Mahalanobis distance from the smooth set:

(4) $$\begin{array}{c} D\left(x_{j}\right)=\sqrt{{\left(x_{j}-\mu \right)}^{T}\Sigma^{-1}\left(x_{j}-\mu \right)}, \end{array}$$

where *x*_*j*_ is the coordinate of a testing feature point, Σ is the covariance matrix of feature points in smooth set, and *μ* is the mean of feature points in the smooth set. As shown in Figure 4(b) the proposed feature distance faithfully captures the anisotropic feature regions.

The feature distance measure has two functions in our algorithm: one is to provide a distribution model in the feature detection step; the other is to control the normal vector filtering process.

### 4.2. Feature Region Segmentation

Feature region detection is commonly solved by thresholding some attributes of mesh surfaces. For example, approaches in [17, 18] employ the normal variance in the local neighborhood as the attribute. However, this scheme involves multiple user-defined parameters such as the size of the local neighbor, tolerant normal variance, etc. In order to avoid these parameters, we adopt a graph-cut algorithm to separate the feature regions from smooth regions.

**Figure 5:**
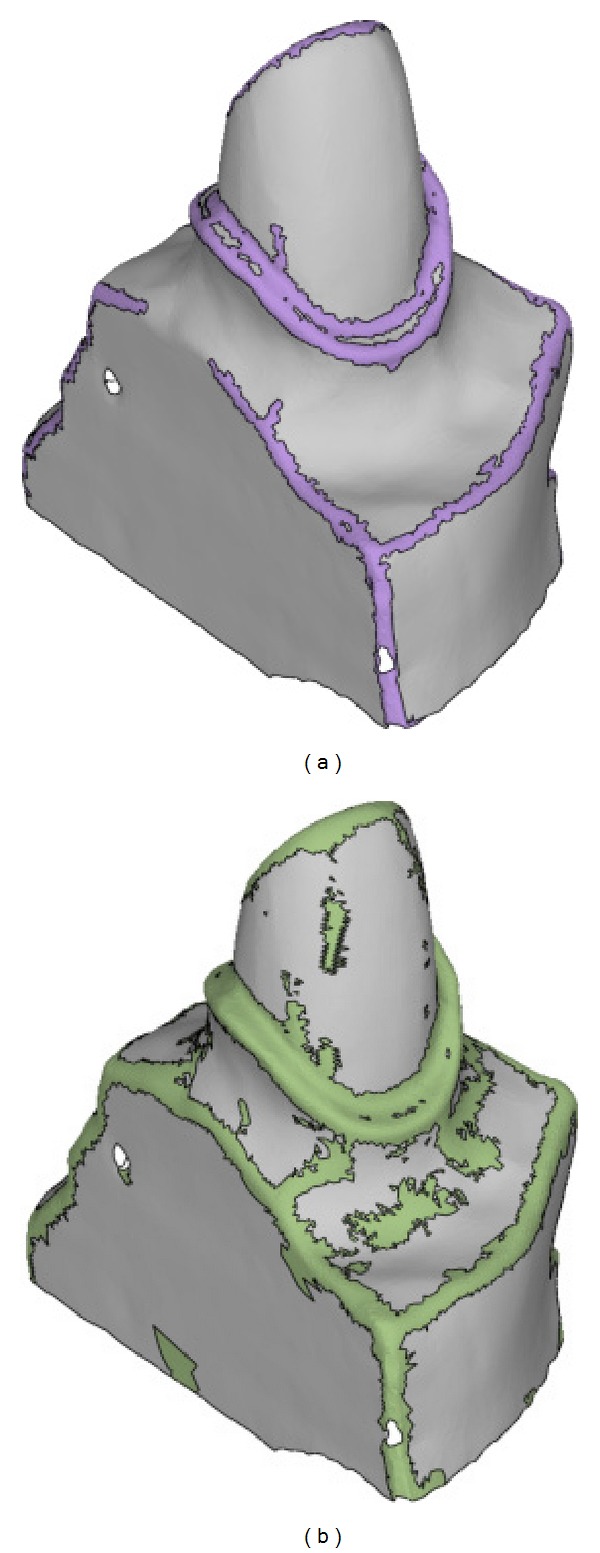
The comparison of feature region detection results: (a) our scheme and (b) the approach based on normal variance proposed by [17, 18]. Our approach provides spatial coherent feature regions. Moreover the thresholding based on different models is avoided.

Let *G*(*F*, *E*, *W*) be the dual graph of *M*(*V*, *F*) where *F* is the nodes of the dual graph, *E* is the edge set of the dual graph, each edge connects two neighboring faces, and *W* is the weights defined on edges. To perform a graph-cut segmentation, we add two virtual nodes. One is the source node which represents the smooth regions; the other is the sink node which represents the feature regions. Then the energy function of the graph-cut segmentation is defined as

(5) $$\begin{array}{c} E\left(S\right)=\lambda R\left(S\right)+B\left(S\right), \end{array}$$

where *S* = {*s*_*i*_ | *i* = 1,2,…, |*S*|} is a labeling for triangles of *M*, *R*(*S*) is the regional penalty for assigning labels, *B*(*S*) is the boundary penalty for assigning different labels between neighbor triangles, and *λ* is the relative importance of the two terms in (5) which is specified as 1.0. The behaviors of the segmentation depend on the definitions of *R*(*S*) and *B*(*S*). To separate feature regions, we define them as follows:

(4.2) $$\begin{array}{c} R\left(s_{i}\right)=\{\begin{array}{cc} D\left(x_{i}\right), & s_{i}=\text{sink,}\\ \frac{1.0}{D\left(x_{i}\right)}, & s_{i}=\text{source,} \end{array}\\ B\left(s_{i},s_{j}\right)=\{\begin{array}{cc} \exp\left(-\frac{||D\left(x_{i}\right)-D\left(x_{j}\right)||}{D_{\max}}\right), & s_{i}\neq s_{j},\\ 0, & s_{i}=s_{j}, \end{array} \end{array}$$

where *D*_max⁡_ is the maximal distance of feature points. We employ the algorithm in [32] to optimize the energy defined in (5). The computation is efficient and the spatial coherence is guaranteed as shown in Figure 5.

## 5. Normal Filtering in a Controllable Manner

**Figure 6:**
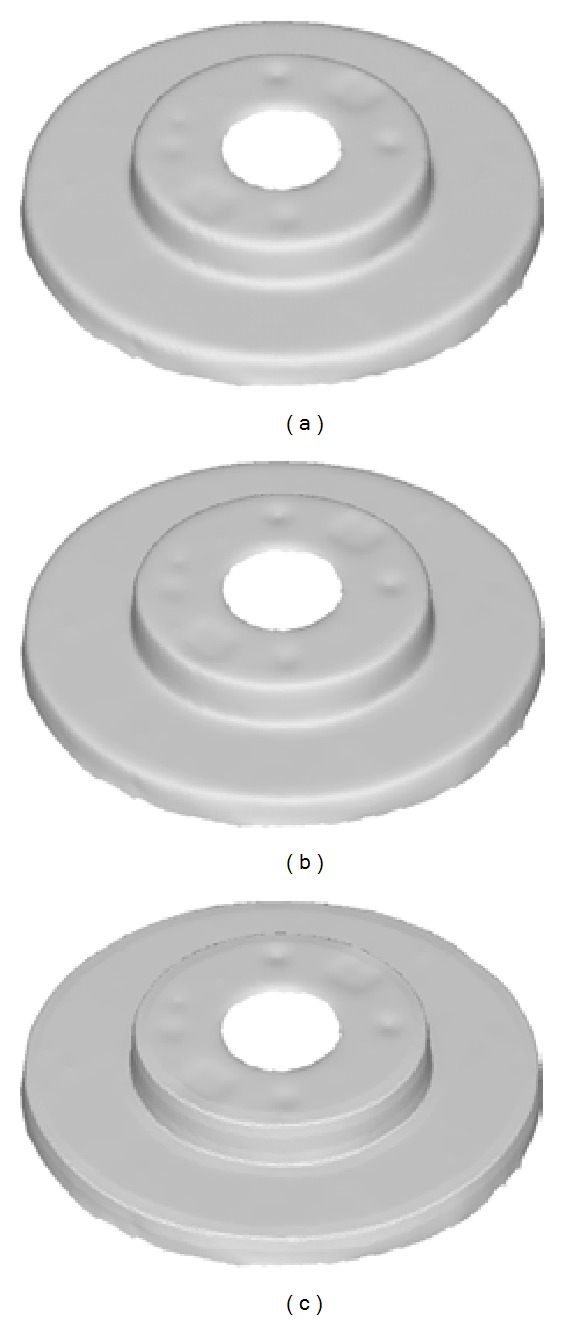
The impact of different values of the parameter *α*: (a) the input of disk model, (b) *α* = 0.2, and (c) *α* = 0.8.

In the previous section, we have confined the following normal filtering in the feature regions so that unnecessary computations are avoided. To reconstruct sharp edge features, the common strategy is to propagate geometry from smooth regions to feature regions; the difference from previous approaches is the way to predict vertex positions. However, these approaches all result in oversharpening geometry since the filtered geometry is the same with the smooth regions where the propagation begins. In contrast, we adopt the feature distance measure defined in (4) to control the normal filtering process:

(7) $$\begin{array}{c} n_{i}^{\prime }=\underset{k\in N_{f_{i}}}{\sum }w_{k}n_{k}, \end{array}$$

where *N*_*f*_*i*__ is the triangles in the one-ring neighborhood of *f*_*i*_. The feature distance weights *w*_*k*_ make the triangles at feature region tend to maintain its original normal vectors, which is defined as

(8) $$\begin{array}{c} w_{k}=\exp\left(-\alpha \cdot \max\left(D\left(x_{1}^{k}\right),D\left(x_{2}^{k}\right),D\left(x_{3}^{k}\right)\right)\right), \end{array}$$

where *x*_1_^*k*^, *x*_2_^*k*^, and *x*_3_^*k*^ are positions of the three vertices of *f*_*k*_. The parameter *α* controls the sharpness of the edge feature region. A larger value of *α* corresponds to a high degree of the sharpness. The impact of different values of parameter *α* is demonstrated in Figure 6. For processing tooth preparation models, the parameter is experimentally chosen as 0.5 in our tests.

In order to propagate the geometry of the smooth region to feature region, we adopt a greedy process to iteratively filter the face normal vectors using (7). The priority is determined by the feature distance measure. After the desired faces normal vectors have been obtained, we update the vertex positions through least square approximation to the filtered normal vectors. We adopt the energy function used in [33]:

(9) $$\begin{array}{c} E_{1}\left(X\right)=\overset{}{\underset{k\in F}{\sum }}\;\overset{}{\underset{\left(i,j\right)\in \partial F_{k}}{\sum }}{\left(n_{i}^{\prime }\cdot \left(x_{i}-x_{j}\right)\right)}^{2}, \end{array}$$

where *X* is the vertex positions. We solve (9) using gradient descent method.

## 6. Results

**Figure 7:**
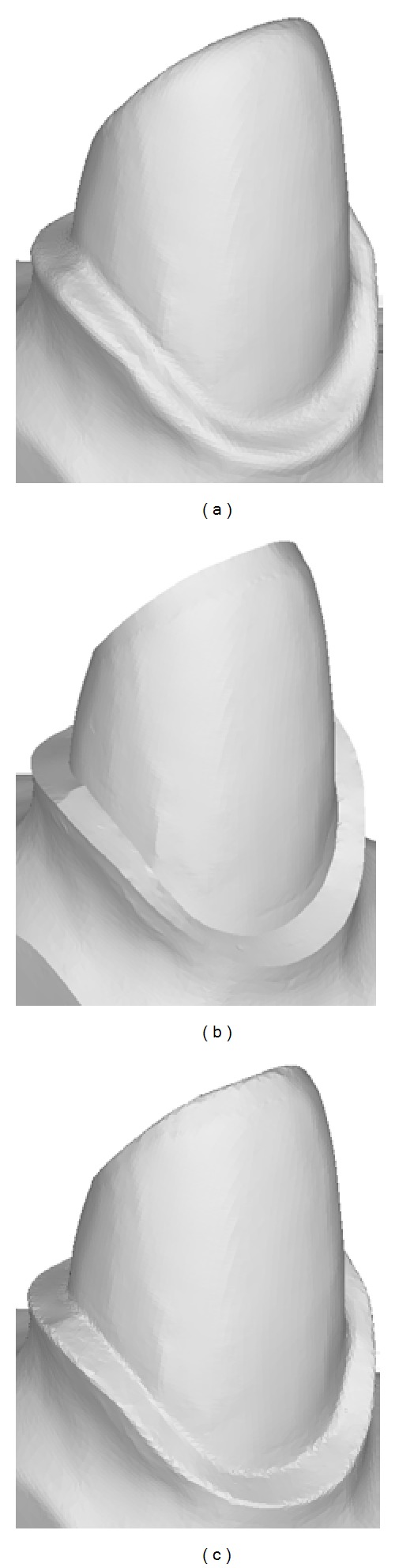
The comparison of sharpening results on a tooth preparation model: (a) the original tooth preparation model of which the region including marginal cervical line is blurred, (b) the result of the method in [18] where the region including marginal cervical is too sharp, and (c) our result enhances the edge feature region and avoids the oversharpening geometry.

**Figure 8:**
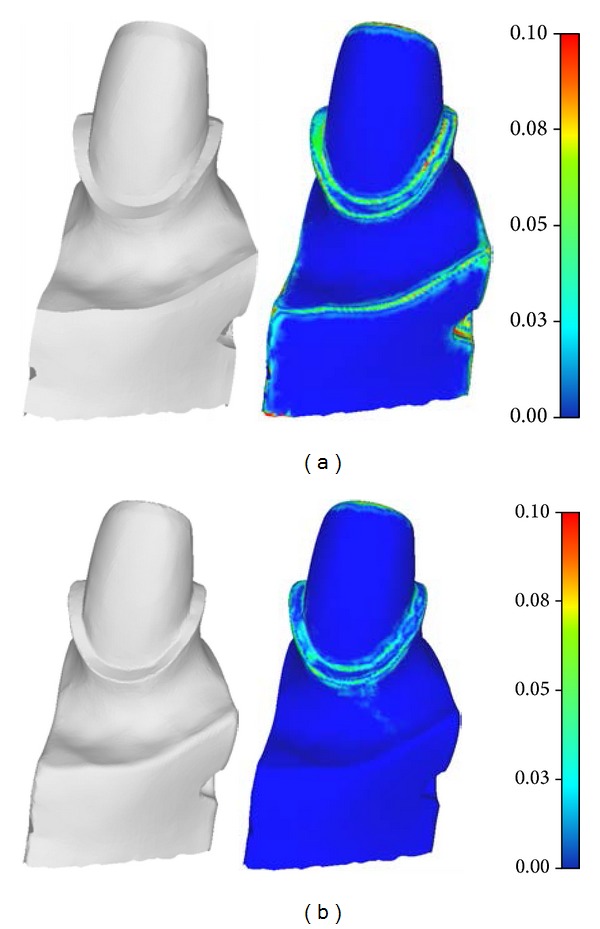
The deviations between the original scanned tooth preparation and its sharpened versions: (a) the sharpened tooth preparation model generated by the method in [18] has relatively large deviations at the regions containing cervical line and (b) our approach maintains the shape of the cervical line in a controllable manner.

We have developed an implementation of the proposed mesh sharpening algorithm using C++ language. We present several tests on tooth preparation and mechanical and arts models below. All tests are conducted on a PC with Intel Core i5 CPU, 2 GB main memory, and Windows XP operating system. We compare our method with the most similar approach [18] which also employs normal filtering. Firstly, we present the results on tooth preparation models. As shown in Figure 7, both our approach and the method in [18] successively enhance the blurred edge features. However, our method avoids the oversharpening geometry which makes the scanned surface unnatural. Specifically, the sharpened edge features generated by the method in [18] are single-edge wide which can be identified through dihedral angles. As for modeling dental restorations, the oversharpening geometry may destroy the original morphology of the cervical lines. We further compare the Hausdorff distance between the original scanned tooth preparation and its sharpened versions generated by the method in [18] and ours. As shown in Figure 8, our controllable sharpening algorithm can maintain the shape of the cervical line while enhancing the regions around it.

**Figure 9:**
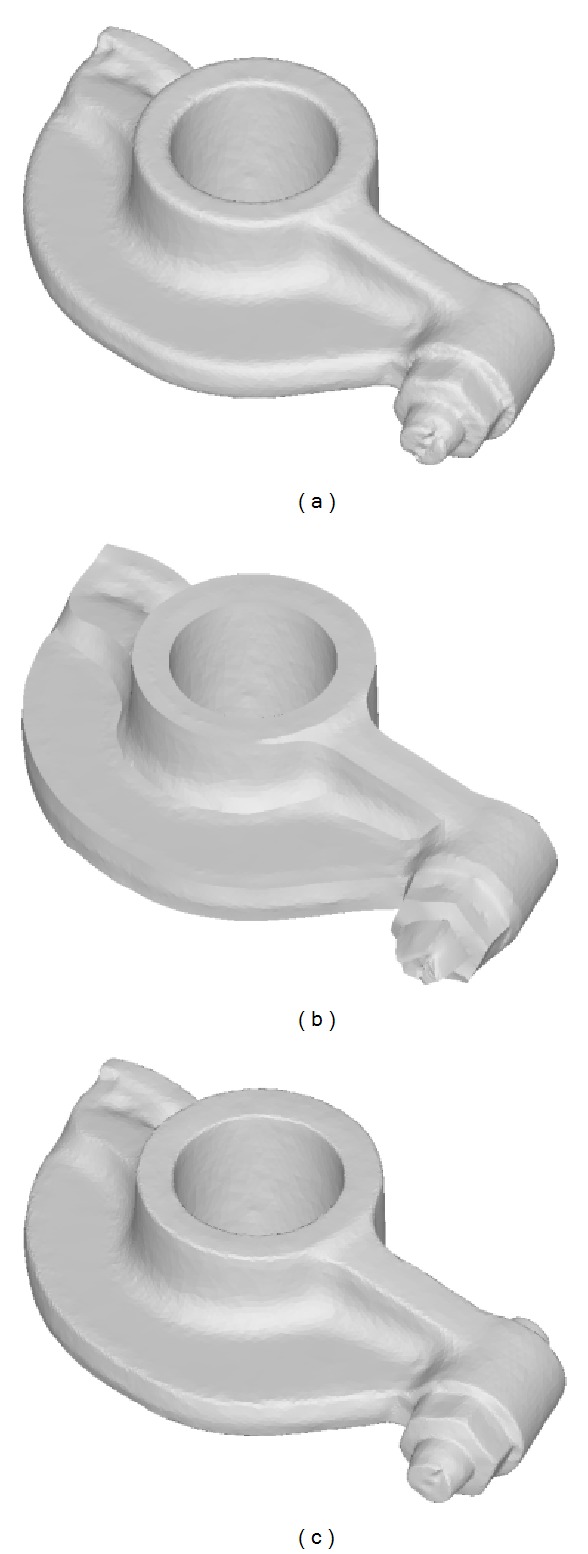
The comparison of sharpening results on the rocker arm model: (a) the original model, (b) the result generated by the method in [18], and (c) the result of our method.

**Figure 10:**
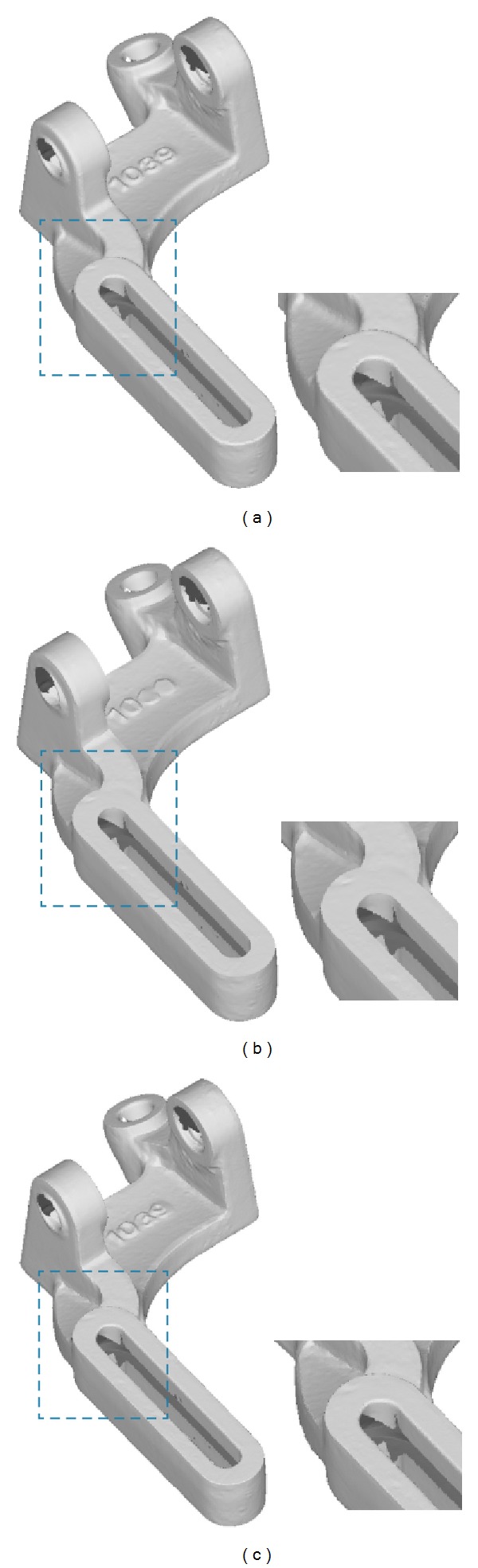
The comparison of sharpening results on a mechanical part model: (a) the original model, (b) the result generated by the method in [18], and (c) the result of our method.

**Figure 11:**
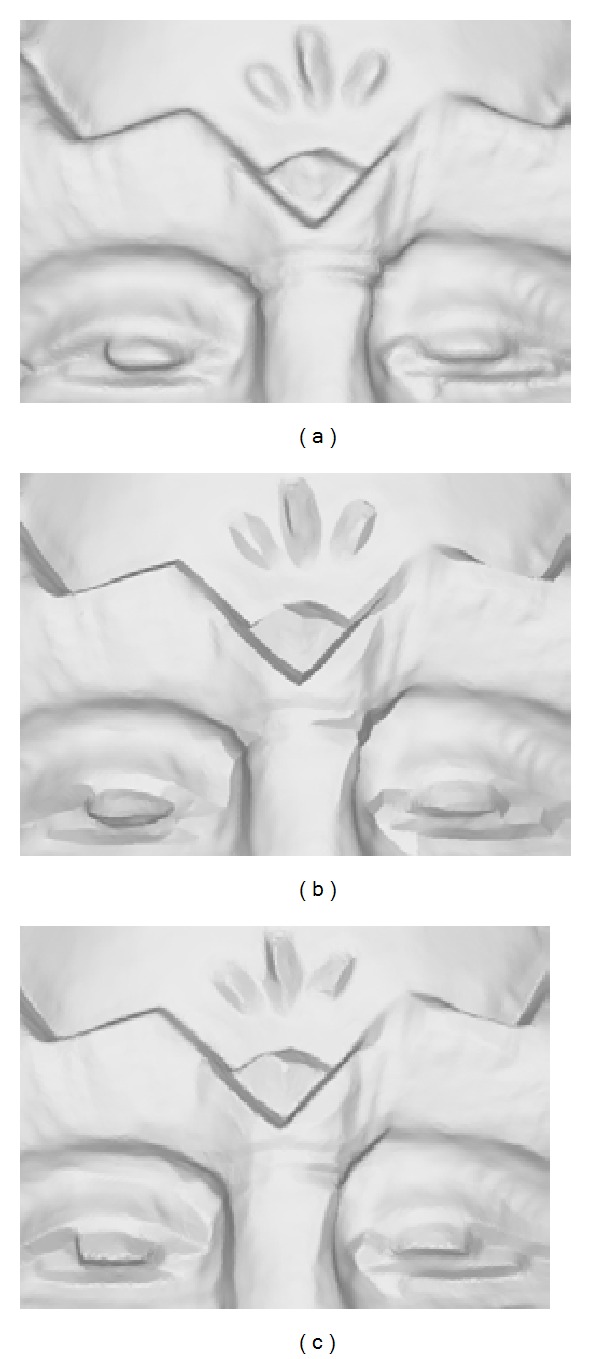
The comparison of sharpening results on a mask model: (a) the original model, (b) the result generated by the method in [18], and (c) the result of our method.

In addition to tooth preparation models, as shown in Figures 9, 10, and 11, our approach is also capable of processing scanned surfaces used in mechanical and arts industry.

Note that prior methods try to directly construct feature lines on mesh surface which can be easily identified through dihedral angles. However, this characteristic is only desired for computer-generated CAD models. For scanned surfaces, the mesh sharpening algorithm should avoid oversharpening geometry. In addition, the scanned models are usually quite large. Therefore, the computational cost is critical for practical applications. The timing statistics of the proposed approach is given in Table 1, from which we can conclude that the time cost is reasonable and approximately linear to the model size.

**Figure 12:**
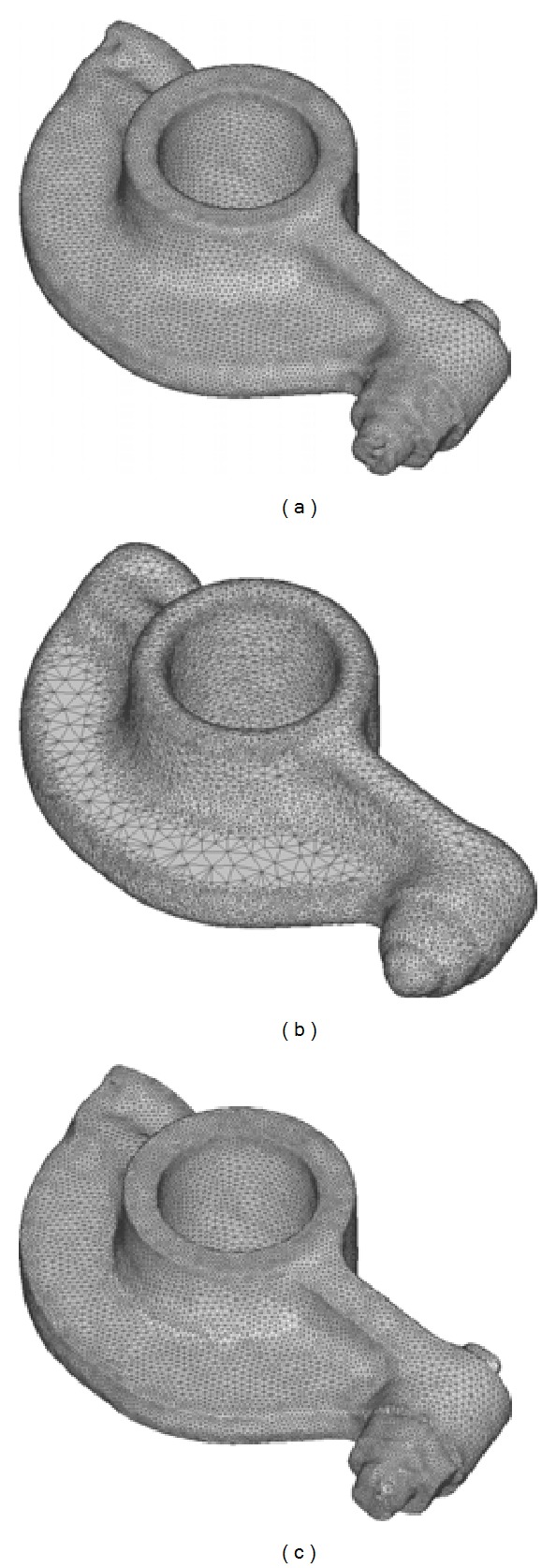
The comparison of the filtering results between the method in [19] and ours: (a) the input rocker arm model, (b) the result generated by the method in [19], and (c) the result of our method. To illustrate the difference, the models are rendered with edges.

We further compare with the mesh enhancing method in [19], which optimizes all vertex positions through moving the vertices in flat regions to high-curvature regions. As shown in Figure 12, all the models have the same number of vertex samples. The result of the method in [19] modifies all the vertex positions, leading to dense sampling in high-curvature regions. In contrast, our result only filters the vertex samples around the edge features. In addition, the time cost of the method in [19] is 196 seconds with our implementation.

## 7. Conclusions

In this paper, we have proposed a novel mesh sharpening algorithm which enhances edge features of scanned surface models in a controllable manner. The main components of the proposed approach consist of two factors: detecting feature regions and propagating the geometry from the smooth regions to the feature regions. By introducing a feature distance measure based on normal tensor analysis, we obtain naturally-enhanced edge features on scanned surfaces like tooth preparation and mechanical and arts models.

## Figures and Tables

**Table 1:** Timing statistics of the proposed approach.

Model (Triangles)	Tooth (41,668)	Rocker arm (41,552)	Part (366,307)	Mask (519,130)
Time cost (second)	9	7	49	74
